# Emergency transapical mitral valve-in-valve implantation for bioprosthesis failure: transapical implantation of an Edwards Sapien-XT in a dysfunctional mitral bioprosthesis in a critical patient

**DOI:** 10.1186/s13019-017-0680-7

**Published:** 2017-12-13

**Authors:** Marco Zanobini, Sabrina Manganiello, Giorgia Bonalumi, Raoul Biondi, Marco Russo, Massimo Mapelli, Francesco Alamanni, Matteo Saccocci

**Affiliations:** 1 Department of Cardiac Surgery, IRCCS - Centro Cardiologico Monzino, Università degli Studi di Milano, Via C. Parea 4, 20138 Milano, Italy; 2 Department of CardioVascular Surgery, Heart Center - University Hospital of Zurich, Zurich, Switzerland; 3 Department of Cardiology -IRCCS - Centro Cardiologico Monzino, Università degli Studi di Milano, Milano, Italy

**Keywords:** Mitral valve stenosis, Bioprosthesis, Transcatheter valve implantation, Valve-in-valve, Transapical, Mitral bioprosthesis, Emergency

## Abstract

**Background:**

Valve-in-Valve (VIV) Transcatheter Aortic Valve Replacement (TAVR) is now the treatment of choice in high-surgical-risk patients with failing aortic bioprosthesis. Although less performed, VIV-Transcatheter Mitral Valve Replacement (TMVR) is a valid treatment option for selected high-risk patients with degenerated mitral bioprostheses. Several cases of elective ViV- TAVR and -TMVR have been reported but only few were performed in critical hemodynamic conditions.

**Case presentation:**

We report the case of a patient underwent balloon-expandable transapical mitral valve-in-valve implantation in an emergency setting due to a severe stenosis of a bioprosthesis in mitral position. The procedure was successfully performed, with no residual mitral regurgitation or paravalvular leaks, and uneventful.

**Conclusion:**

Transcatheter transapical mitral valve-in-valve implantation could represent a feasible and effective strategy even in critical setting.

**Electronic supplementary material:**

The online version of this article (10.1186/s13019-017-0680-7) contains supplementary material, which is available to authorized users.

## Background

Valve-in-Valve (VIV) Transcatheter Aortic Valve Replacement (TAVR) is now the treatment of choice in high-surgical-risk patients with failing aortic bioprosthesis [1]. Although less performed, VIV-Transcatheter Mitral Valve Replacement (TMVR) [2] represents a valid treatment option for selected high-risk patients with degenerated mitral bioprostheses. Several cases of elective ViV- TAVR and -TMVR have been reported, but only a few were performed in critical hemodynamic conditions, especially for dysfunctioning mitral bioprosthesis [3–5]. Over the last decade, the use of bioprosthesis or mitral valve reconstruction, instead of mechanical valves, has shown an important worldwide increase thanks to the improved long-term results and inspired to the desire of avoiding the need of life-long systemic anticoagulation. The durability of bioprostheses, especially in mitral position, can be very variable, depending on patient’s and valve characteristics. Although surgical redo operation is often possible, when there are no specific contraindications, it’s widely known that is accompanied by an increased mortality depending on age, comorbidities and elective or urgent status of the procedure. In this scenario, the incidence of failing surgical valves in high surgical risk patients will surely increase. Even more important is the choice of the right treatment option in patients with critical conditions with high or unacceptable surgical risks. Usually stented bioprosthesis present a progressive deterioration, evaluable with a correct echocardiographic follow-up, permitting a comfortable planning of the procedure but sometimes patients could be admitted directly to the ER in life-threating conditions without previous important symptoms. We report the case of a patient underwent balloon-expandable transapical mitral valve-in-valve implantation in an emergency setting due to a severe mitral stenosis of a surgical bioprosthesis.

## Case presentation

**Fig. 1:**
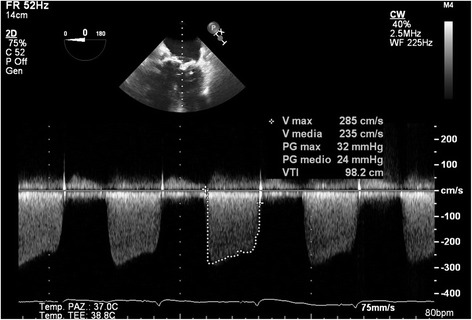
Preoperative transthoracic echocardiography showing severe mitral stenosis (transvalvular mean gradient 24 mmHg)

An 82-year-old woman affected by hypertension, grade 1 obesity, severe chronic obstructive pulmonary disease (COPD), atrial fibrillation (AF) and history of previous cardiac surgery was admitted to the emergency room (ER) for acute pulmonary edema and renal insufficiency. Eight years before she underwent aortic and mitral valve replacement (Magna Aortic 23 mm, Magna Mitral 27 mm – Edwards Lifescience, Irvine, CA, US), tricuspid valve repair and atrial fibrillation radiofrequency ablation complicated by complete AV-block and pacemaker implantation. During the years she remained asymptomatic without signs of heart failure. The trans-thoracic echocardiography (TTE) performed at admission showed no signs of endocarditis, no degeneration of the aortic bioprosthesis, good function of the repaired tricuspid valve but a very severe mitral prosthesis stenosis (mean gradient 24 mmHg, PHT 420 msec, area 0,52 cm^2^; PAPS 66 mmHg) [Fig. 1; Additional file 1: Video 1]. ECG-gated MDCT (multidetector computed tomography) recorded no significant coronary artery disease, calcified thoracic aorta, a calcified mitral bioprosthesis (outer-diameter 28.9mmx28.6 mm, inner-diameter 24.9 × 24.4 mm) and bilateral severe pleural effusion. Laboratory testing showed severe anemia. The patient was initially medically treated with endovenous diuretics, blood transfusions, Continous Veno-Venous Hemofiltration (CVVH) and bilateral pleural drainage. Despite medical therapy optimization, the patient ‘s conditions remained critical with hemodynamic instability, hypotension, initial neurological impairment and acute anuric renal failure. Evaluated morbidity and mortality risk for surgical redo mitral replacement (EuroSCORE II = 19,84; CHA_2_DS_2_-VASC Score = 4; HAS BLED score = 4) our multidisciplinary Heart-Team decided for an emergency ViV-TMVR in general anesthesia with TEE-monitoring.Additional file 1: Video 1.Preoperative TransThoracic Echocardiography. (MP4 4.90 mb)

**Fig. 2:**
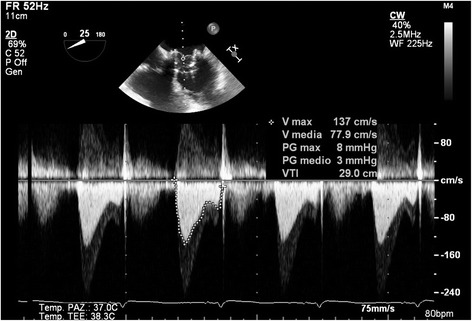
Valve-in-Valve implantation result

**Fig. 3:**
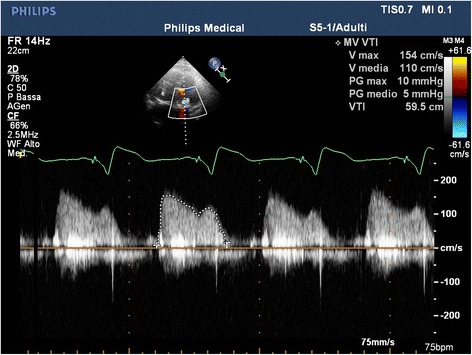
PreDischarge Trans-Thoracic echocardiography Mitral prosthesis’ gradient

**Fig. 4:**
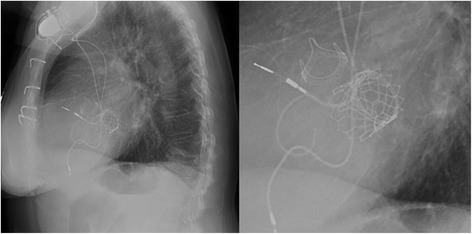
30-days Trans-Thoracic echocardiography Mitral prosthesis’ gradient

A temporary endocardial pacing leads was introduced in the right ventricle through a 6-F sheath positioned into the left femoral vein. Apex position was optimally detected by TTE evaluation and the pericardium was reached through a left anterolateral thoracotomy. As in our standardized technique for transapical approach, we proceed with the removal of all the adherence and with the positioning of 2 perpendicular “U”-stitches (2/0 polypropylene - Prolene; Ethicon, Inc.), reinforced by pledgets, directly on the myocardial tissue of the apex. Performed the transapical puncture, we positioned a 7-F sheath in order to introduce a 0.035 guide-wire through the ventricle toward the mitral prosthesis reaching the pulmonary vein. Over the guide-wire, we inserted a MultiPurpose catheter to exchange the previous wire with an ExtraStiff .035 in. (Cook Medical). The valve delivery system was introduced through an expandable 21-F E-Sheath (Edwards Lifescience – Irvine, CA, US). Under fluoroscopic and TEE guidance, we proceeded with the deployment of a 29 mm Sapien-3 valve (Edwards Lifescience – Irvine, CA, US) using the annulus of the surgical bioprosthesis to recognize the correct position (Fig. 2). There were no paravalvular leaks or mitral regurgitation, and we immediately observed a significant improvement in intraprocedural monitoring parameter. The procedure was uneventful, as well as ICU course (no needs of CVVH, inotropes or prolonged mechanical ventilation). The patient was discharged 10 days after ViV-TMVR in good clinical condition (NYHA class I-II). At pre-discharge TTE transvalvular mitral mean gradient was 3 mmHg, PAPS decreased to 29 mmHg and LV Ejection Fraction remained 55% Fig. 3, Additional file 2: Video 2, Additional file 3: Video 3. At 1 month follow-up, the patient was asymptomatic (NYHA class I-II) and the TTE confirmed the absence of valve dysfunction or Paravalvular leak [Fig. 4]. Additional file 2: Video 2.Postoperative TransThoracic Echocardiography. (MP4 5.45 mb)Additional file 3: Video 3.Postoperative TransThoracic Echocardiography. (MP4 2.30 mb)

## Discussion and conclusions

Degeneration of surgically implanted bioprosthesis in elderly patients is an increasing problem due to the longer life-expectancy. Moreover, this issue is going to interest also a younger population due to the growing percentage of bioprosthesis implantation instead of the mechanical ones. According to the current literature, the perioperative risk of redo surgical intervention may reach high percentage, up to 15% for redo aortic replacement, depending on age, clinical conditions and comorbidities. To this increasing number of patients, we should add the several situations in which surgery can’t be performed because of technical contraindications (porcelain aorta, really calcified mitral annulus, multiple redos, frailty, etc.). We are in an era where TAVR is already the therapy of choice for high-risk patients, and in the next year, its indications are moving to the moderate-risk ones. The valve-in-valve concept was developed by Walther et al. in 2007 with the intent of decrease the reoperative risk in patients with a dysfunctional bioprosthesis. ViV-TAVR is nowadays a standard procedure to treat elderly patients with degenerated bioprosthesis, while mitral valve-in-valve implantation is not yet so frequent probably due to the difficulties of reaching the mitral with a peripheral approach. The need of a transapical puncture, as a unique option, is probably going to end with the expanding possibilities linked to transeptal system development. Technically, the deployment of a transcatheter valve into a surgically implanted bioprosthesis is a quite simple procedure for skilled operators thanks to the possibility to use the annulus of the surgical prosthesis as a reference point for the deployment of the new one. Furthermore, using the degenerated valve as a landing zone for the implantation assure a good stability of the transcatheter prosthesis and it’s usually not accompanied by significant paravalvular leaks. Transapical approach requests high surgical skills, but with the actual low-profile introducer sheaths the risk of complications is minimized.

According to our experience, transapical valve-in-valve transcatheter mitral implantation can be a feasible and safe way to treat bioprosthesis dysfunction even in urgent and emergency settings, as we have shown in this case.

## Abbreviations

AF: Atrial Fibrillation
AV block: atrio-ventricular block
COPD: Chronic Obstructive Pulmonary Disease
CVVH: Continuous Veno-Venous Hemofiltration
ECG-gated MDCT: Electrocardiographic-gated Multiple Detector Computed Tomography
ER: Emrgency Room
LV: Left Ventricle
NYHA: New York Heart Association
PM: Pacemaker
TAVR: Transcatheter Aortic Valve Replacement
TEE: Trans-Esophageal Echocardiography
TMVR: Valve-in-valve Transcatheter Mitral Valve Replacement
TTE: Trans-Thoracic Echocardiography
VIV: Valve-in-Valve
